# Transformation of t(14;18)-negative follicular lymphoma to plasmablastic lymphoma: a case report with analysis of genetic evolution

**DOI:** 10.1186/s13000-024-01512-2

**Published:** 2024-06-22

**Authors:** Sojung Lim, Jiwon Koh, Jeong Mo Bae, Hongseok Yun, Cheol Lee, Jin Ho Paik, Tae Min Kim, Yoon Kyung Jeon

**Affiliations:** 1 Department of Pathology, Seoul National University Hospital, Seoul National University College of Medicine, 101 Daehak-Ro, Jongno-Gu, Seoul, 03080 Republic of Korea; 2 Department of Genomic Medicine, Seoul National University Hospital, Seoul National University College of Medicine, 101 Daehak-Ro, Jongno-Gu, Seoul, 03080 Republic of Korea; 3 Department of Pathology, Seoul National University Bundang Hospital, Seoul National University College of Medicine, 82 Gumi-Ro 173 Beon-Gil, Bundang-Gu, Seongnam-Si, Gyeonggi-Do 13620 Republic of Korea; 4 Department of Internal Medicine, Seoul National University Hospital, Seoul National University College of Medicine, 101 Daehak-Ro, Jongno-Gu, Seoul, 03080 Republic of Korea; 5 Cancer Research Institute, Seoul National University, 101 Daehak-Ro, Jongno-Gu, Seoul, 03080 Republic of Korea

**Keywords:** *BCL2*-rearrangement−negative follicular lymphoma, Plasmablastic lymphoma, Histological transformation, Clonal evolution, Targeted gene sequencing

## Abstract

**Background:**

Follicular lymphoma (FL) is characterized by t(14;18)(q32;q21) involving the *IGH* and *BCL2* genes. However, 10–15% of FLs lack the *BCL2* rearrangement. These *BCL2*-rearrangement−negative FLs are clinically, pathologically, and genetically heterogeneous. The biological behavior and histological transformation of such FLs are not adequately characterized. Here, we report the first case of t(14;18)-negative FL that rapidly progressed to plasmablastic lymphoma (PBL).

**Case presentation:**

A previously healthy 51-year-old man presented with leg swelling. Computed tomography (CT) showed enlarged lymph nodes (LNs) throughout the body, including both inguinal areas. Needle biopsy of an inguinal LN suggested low-grade B-cell non-Hodgkin lymphoma. Excisional biopsy of a neck LN showed proliferation of centrocytic and centroblastic cells with follicular and diffuse growth patterns. Immunohistochemical analysis showed that the cells were positive for CD20, BCL6, CD10, and CD23. BCL2 staining was negative in the follicles and weak to moderately positive in the interfollicular areas. *BCL2* fluorescence in situ hybridization result was negative. Targeted next-generation sequencing (NGS) revealed mutations in the *TNFRSF14*, *CREBBP*, *STAT6*, *BCL6*, *CD79B*, *CD79A*, and *KLHL6* genes, without evidence of *BCL2* or *BCL6* rearrangement. The pathologic and genetic features were consistent with t(14;18)-negative FL. Two months after one cycle of bendamustine and rituximab chemotherapy, the patient developed left flank pain. Positron emission tomography/CT showed new development of a large hypermetabolic mass in the retroperitoneum. Needle biopsy of the retroperitoneal mass demonstrated diffuse proliferation of large plasmablastic cells, which were negative for the B-cell markers, BCL2, BCL6, and CD10; they were positive for MUM-1, CD138, CD38, and C-MYC. The pathologic findings were consistent with PBL. The clonal relationship between the initial FL and subsequent PBL was analyzed via targeted NGS. The tumors shared the same *CREBBP*, *STAT6*, *BCL6*, and *CD79B* mutations, strongly suggesting that the PBL had transformed from a FL clone. The PBL also harbored *BRAF* V600E mutation and *IGH*::*MYC* fusion in addition to *IGH*::*IRF4* fusion.

**Conclusions:**

We propose that transformation or divergent clonal evolution of FL into PBL can occur when relevant genetic mutations are present. This study broadens the spectrum of histological transformation of t(14;18)-negative FL and emphasizes its biological and clinical heterogeneity.

## Background

Follicular lymphoma (FL) is an indolent B-cell lymphoma characterized by t(14;18)(q32;q21) involving the *IGH* and *BCL2* genes in 85–90% of patients [1]. There are also recurrent mutations of epigenetic regulator genes, genes involved in JAK-STAT or BCR-NF-κB signaling pathways, *TNFRSF14*, and *BCL6* [2, 3]. However, 10–15% of FLs lack the characteristic *BCL2* rearrangement. These *BCL2*-rearrangement−negative FLs are clinically, pathologically, and genetically heterogeneous. Cases that are otherwise genetically similar to t(14;18)-positive FLs have been reported, with loss of 1p36 or mutations in *TNFRSF14* and epigenetic regulator genes. There have also been cases with *STAT6* and *CREBBP* co-mutations and less frequent mutations in *TNFRSF14* or *EZH2* [3, 4]. Clinically, some patients present with low-stage disease predominantly involving the inguinal lymph nodes (LNs). These cases frequently show a diffuse pattern with CD23 expression, and *STAT6* and *CREBBP* co-mutations are common [3, 4]. There are also cases harboring *BCL6* rearrangements, which tend to present at a higher stage and histologic grade [4].

Although initially treatment-responsive, FL is considered incurable and eventually relapses or transforms into an aggressive lymphoma. Histological transformation of FL occurs in 25–35% of cases, most often as diffuse large B-cell lymphoma (DLBCL) [1]. Transformation into high-grade B-cell lymphoma with *MYC* and *BCL2* rearrangements, B-lymphoblastic leukemia/lymphoma, classic Hodgkin lymphoma, and plasmablastic lymphoma (PBL) has also been reported [1, 5]. Histological transformation is generally accompanied by the activation of oncogenes such as *MYC* and inactivation of tumor suppressor genes such as *TP53* and *CDKN2A* [2].

Transformation of FL into PBL is rare, with only a few reported cases, all of which were identified in *BCL2*-rearranged FLs [5–9]. By contrast, histological transformation of t(14;18)-negative FL is less clearly characterized, and transformation to PBL has not been reported. Here, we present the first case of plasmablastic transformation in a t(14;18)-negative FL, along with an analysis of its genetic evolution.

## Case presentation

A previously healthy 51-year-old man presented complaining of leg swelling for 2 months. Physical examination revealed lymphadenopathies in both inguinal areas. Computed tomography (CT) showed enlarged LNs throughout the body above and below the diaphragm. The serum lactate dehydrogenase (LDH) level was mildly elevated at 241 (normal, 100–225) IU/L.

Needle biopsy of a right inguinal LN revealed diffuse proliferation of small to intermediate-sized lymphocytes with diffuse, strong expression of CD20 and CD23. The cells were also focally positive for BCL2, BCL6, and CD10. Pathologic findings were consistent with low-grade B-cell non-Hodgkin lymphoma, but the diagnosis was inconclusive.

**Fig. 1:**
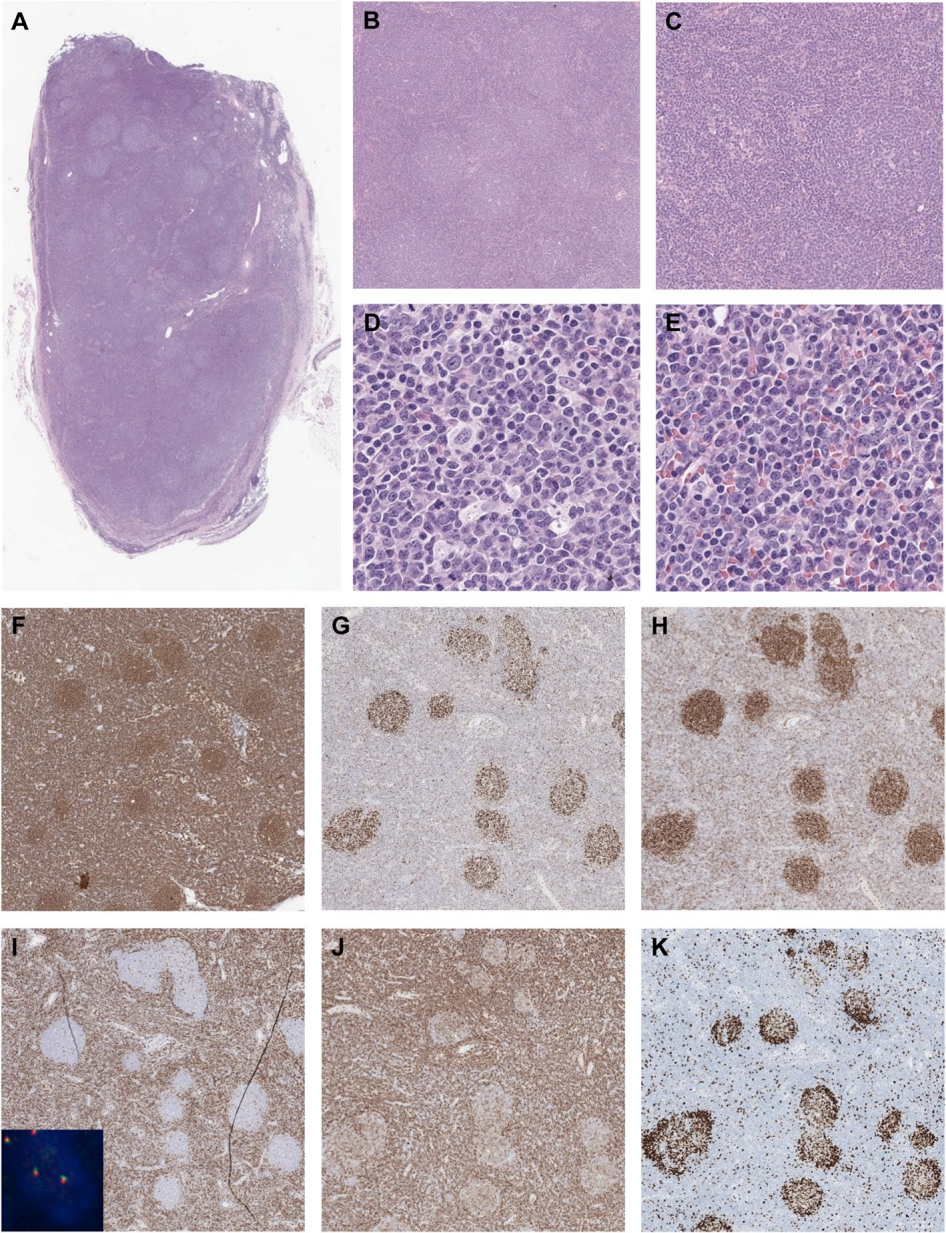
Pathological findings of follicular lymphoma in the neck lymph node. **A **−** C** H&E shows mixed **B** follicular and **C** diffuse growth patterns. **D**, **E** Higher magnification shows centrocytic and centroblastic proliferation in **D** follicular and **E** diffuse interfollicular areas. **F** CD20, **G** BCL6, and **H** CD10 are positive in both the follicular and interfollicular areas. **I** BCL2 is negative in the follicles and weak to moderately positive in the interfollicular areas (Inset: The *BCL2* translocation was negative on break-apart FISH). **J** CD23 shows diffuse, strong expression in the interfollicular areas. CD23 expression is weaker in the follicles with a retained follicular dendritic cell meshwork. **K** The Ki-67 proliferation index is 70% in the follicles and 7% in the interfollicular areas. (Original magnification × 12.5 for A; × 40 for B, F − K; × 100 for C; × 400 for D, E)

An excisional biopsy of a right neck LN was subsequently performed; centrocytic and centroblastic proliferation with follicular and diffuse growth patterns was observed (Fig. 1A − E). The follicular dendritic cell meshwork was retained in the follicular areas, according to the results of CD21 immunohistochemistry. CD20, BCL6, CD10, and CD23 staining results were positive in both the follicular and interfollicular areas. BCL2 staining results were negative in the follicles and weak to moderately positive in the interfollicular areas. The Ki-67 proliferation index was 7% in the interfollicular areas and 70% in the follicles (Fig. 1F − K). Cyclin D1, SOX11, and MUM1 staining results were negative. Histologically, t(14;18)-negative FL was suspected, but other low-grade B-cell lymphomas needed to be ruled out.

**Fig. 2:**
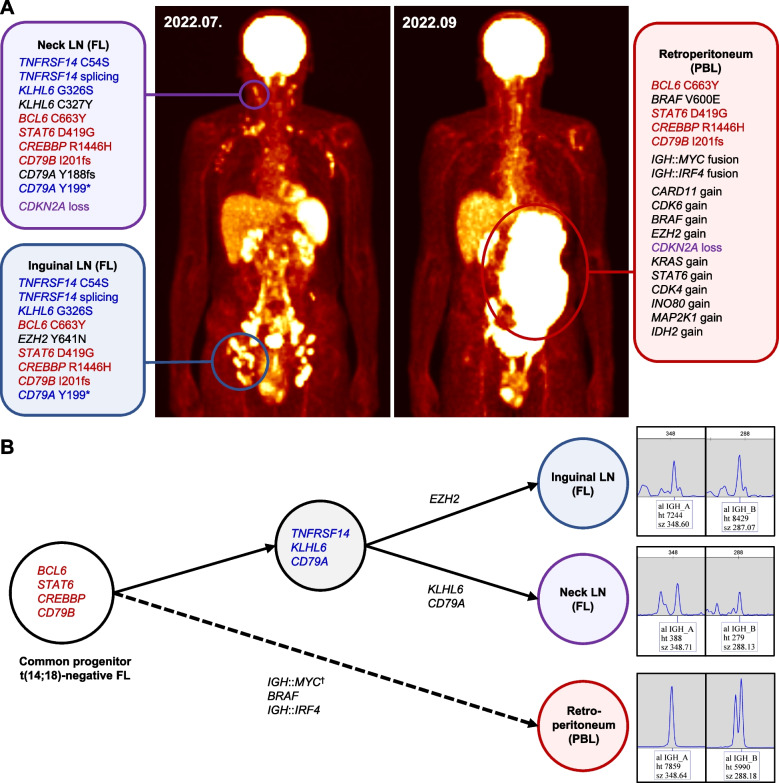
Mutational profiles of the follicular lymphoma and plasmablastic lymphoma.  **A** Initial fluorine-18 labeled fluorodeoxyglucose positron emission tomography/computed tomography (FDG PET/CT) taken on July 2022 showed hypermetabolic lymph nodes in the bilateral neck, axillae, aortocaval, iliac, and inguinal areas. On FDG PET/CT taken in September 2022 after one cycle of chemotherapy, the hypermetabolism in the lymph nodes had decreased, but a new mass had developed in the left retroperitoneum. Targeted next-generation sequencing for FL in the neck and inguinal lymph nodes showed many overlapping mutations. However, the PBL had a *BRAF* V600E mutation, *IGH*::*MYC* fusion, *IGH*::*IRF4* fusion, and copy number gains in multiple genes, in addition to the mutations shared with FL. **B** The mutation profiles suggest that the PBL had transformed from a t(14;18)-negative FL harboring *BCL6*, *STAT6*, *CREBBP*, and *CD79B* mutations. The *IGH* gene rearrangement test showed the same clones in all three specimens, supporting the derivation of the FL and PBL from a common progenitor. ^✝^*IGH*::*MYC* fusion was detected only in PBL on NGS. *IGH*::*MYC* fusion was not detected in FL of the neck LN, but *MYC* translocation was observed in some FL cells on break-apart FISH of the neck LN. *IGH*::*MYC* fusion in FL of the inguinal LN was detected at a very low frequency; thus, it was filtered out on NGS because of insufficient evidence. (Red indicates mutations shared in all three samples, blue indicates mutations shared in the two FL samples, and purple indicates copy number alterations shared between samples. Abbreviations: FL, follicular lymphoma; LN, lymph node; PBL, plasmablastic lymphoma)

Targeted next-generation sequencing (NGS) of DNA and/or RNA (121 genes) involved in the pathogenesis of lymphoid neoplasms was performed for diagnostic purposes [10]. Mutations in the *TNFRSF14* (C54S and splicing mutation), *CREBBP* (R1446H in the histone acetyltransferase domain), *STAT6* (D419G in the DNA binding domain), *BCL6* (C663Y in the zinc finger domain), *CD79B* (I201fs in the immunoreceptor tyrosine-based activation motif (ITAM) domain), *CD79A* (Y188fs and Y199* in the ITAM domain), and *KLHL6* (G326S and C327Y) genes were found (Fig. 2A). No fusion genes were detected; *BCL2* and *BCL6* break-apart fluorescence in situ hybridization (FISH) results were also negative (Fig. 1I). The pathologic features and mutation profile were consistent with t(14;18)-negative FL (i.e., FL with a predominantly diffuse growth pattern [dFL] in the 5th Edition of the World Health Organization Classification of Haematolymphoid Tumours and *BCL2*-rearrangement−negative, CD23-positive follicle center lymphoma [*BCL2-R*−negative CD23^+^ FCL] in the International Consensus Classification of Mature Lymphoid Neoplasms) [3, 11, 12].

Fluorine-18 labeled fluorodeoxyglucose (FDG) positron emission tomography (PET)/CT showed multiple hypermetabolic LNs above and below the diaphragm, and bone marrow involvement was confirmed on biopsy (Fig. 2A). The patient was diagnosed with stage IV FL and received one cycle of bendamustine and rituximab chemotherapy.

Two months after chemotherapy, the patient developed left flank pain. The serum creatinine and uric acid levels were elevated at 1.86 (normal, 0.7–1.4) and 10.6 (normal, 3.0–7.0) mg/dL, respectively. FDG PET/CT showed that the hypermetabolic LNs had disappeared, but a new hypermetabolic mass diffusely involving the left kidney and retroperitoneum had developed (Fig. 2A). Whereas the maximum standardized uptake values (SUV_max_) of the LNs in the initial FDG PET/CT examination were 9.1 for the inguinal LNs and 3.9 for the neck LNs (Fig. 2A, left), the SUV_max_ of the retroperitoneal mass was 21.6 (Fig. 2A, right). The serum LDH level was elevated at 682 IU/L.

**Fig. 3:**
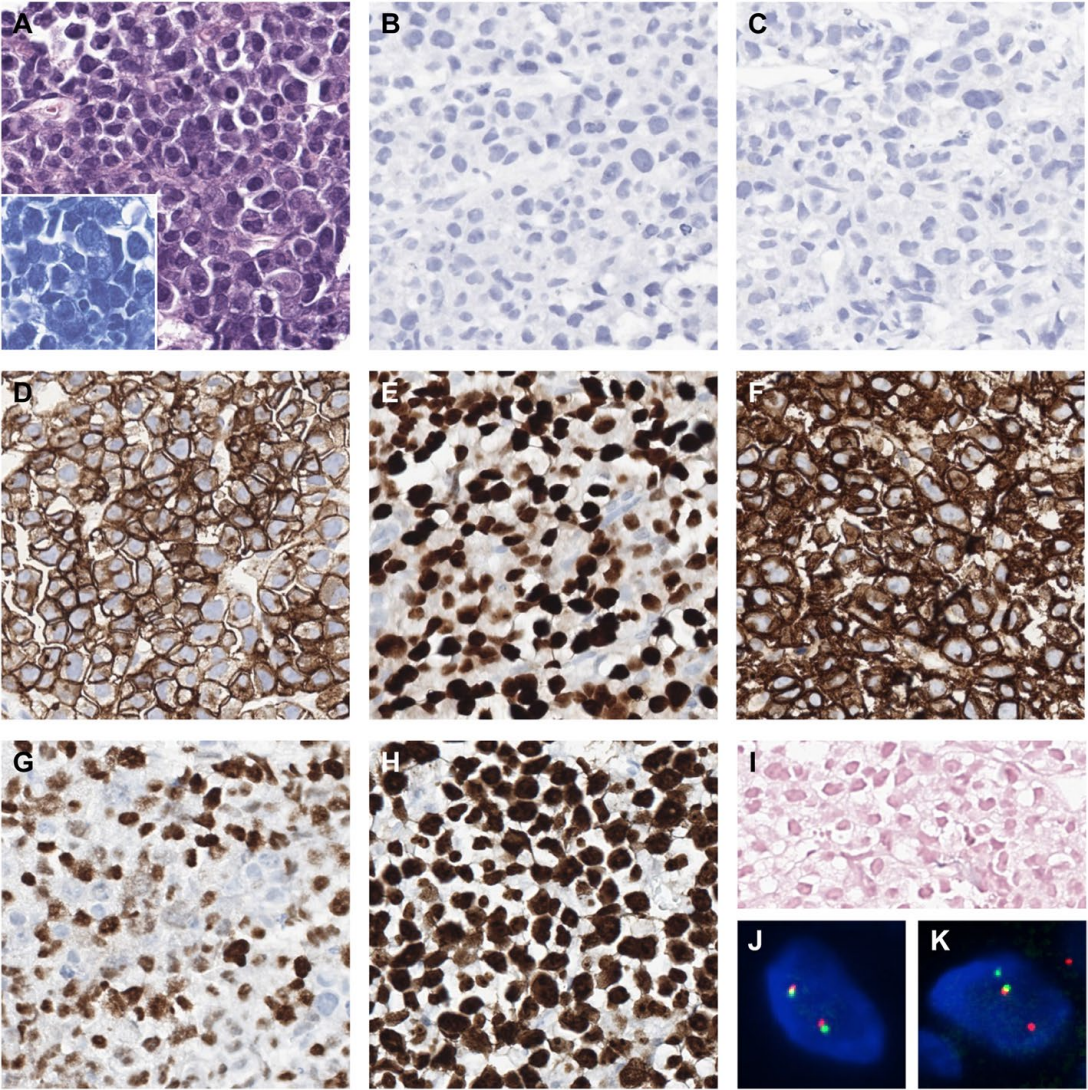
Pathological findings of plasmablastic lymphoma in the retroperitoneum.  **A** H&E shows diffuse proliferation of plasmablastic cells (Inset: Giemsa stain). B-cell markers **B** CD20 and **C** CD79a are both negative. **D** CD43, **E** MUM-1, and **F** CD138 are positive. **G** C-MYC is positive in 85% of the cells. **H** The Ki-67 proliferation index is > 95%. **I** EBER in situ hybridization is negative. **J** The *BCL2* translocation was negative on break-apart FISH. **K** The *MYC* translocation was positive on break-apart FISH. (Original magnification × 400 for A − I)

Progression of FL was suspected, and needle biopsy of the retroperitoneal mass was performed. Diffuse proliferation of large plasmablastic cells with eccentric nuclei and prominent nucleoli was observed, along with a minor component of intermediate-sized plasmacytoid cells (Fig. 3A). B-cell markers CD20, CD79a, and PAX-5 were negative (Fig. 3B, C), as were BCL2, BCL6, CD10, ALK, and HHV-8 LANA-1. CD43, MUM-1, CD138, and CD38 were positive, C-MYC was positive in 85% of cells, and the Ki-67 proliferation index exceeded 90%. Epstein-Barr virus (EBV)-encoded RNA (EBER) in situ hybridization results were negative (Fig. 3D − I). XBP1 and CD56 were also negative. The overall pathologic findings were consistent with PBL.

Targeted NGS was performed on the retroperitoneal PBL and the inguinal LN biopsy specimens (Fig. 2A). The inguinal FL shared the same *TNFRSF14*, *CREBBP*, *STAT6*, *BCL6*, *CD79B*, *CD79A* (Y199*), and *KLHL6* (G326S) mutations with the FL in the neck LN. The *CD79A* Y188fs mutation detected in the neck LN was also detected in the inguinal LN, but it was not reported due to low coverage. By contrast, *EZH2* Y641N (according to RefSeq NM_001203247, or Y646N according to RefSeq NM_004456), a hotspot mutation in the SET domain of *EZH2*, was detected only in the inguinal LN, and *KLHL6* C327Y was detected only in the neck LN. Interestingly, the PBL also harbored identical *CREBBP*, *STAT6*, *BCL6*, and *CD79B* mutations to the FL. In addition, *BRAF* V600E mutation, *IGH*::*MYC* fusion, and *IGH*::*IRF4* fusion were detected by NGS. *MYC* translocation was confirmed by break-apart FISH, with translocation in more than 50% of cells (Fig. 3K). Although NGS did not detect *IGH*::*MYC* fusion in FL of the neck LN, FISH revealed *MYC* translocation in some cells. *IGH*::*MYC* fusion was detected by NGS in FL of the inguinal LN but at a very low frequency; thus, it was filtered out because of insufficient evidence. By contrast, *IRF4* translocation in FL was not detected by either NGS or FISH. There were also multiple copy number alterations, including copy number gains (up to 4 to 5 copies) in the *CARD11*, *CDK6*, *BRAF*, *EZH2*, *KRAS*, *STAT6*, *CDK4*, *INO80*, *MAP2K1*, and *IDH2* genes and a copy loss of *CDKN2A* in the PBL. These results were not shown in the original FL except for the *CDKN2A* loss, which was also detected in the neck LN. The mutation profile suggested that the PBL had transformed from the t(14;18)-negative FL. The clonal relationship between FL and PBL was further supported by the detection of the same clones on the IdentiClone® *IGH* Gene Clonality Assay (Invivoscribe Technologies) (Fig. 2B).

The patient was subsequently given etoposide, prednisolone, vincristine, cyclophosphamide, and doxorubicin for PBL. However, he developed septic shock; the disease ultimately progressed, and the patient died of disease 6 months after the diagnosis of FL and 3 months after the diagnosis of PBL.

## Discussion and conclusions

Although the histological transformation of FL is a well-known phenomenon, it mostly involves the transformation of t(14;18)-positive FL into DLBCL of the germinal center B-cell subtype; transformation into PBL is rare [1]. PBL is a rare B-cell lymphoma mostly reported in the oral cavity of human immunodeficiency virus (HIV)-positive or immunosuppressed patients [1, 13]. Association with EBV is documented in 60–75% of patients; association with EBV is negative mostly in HIV-negative patients [13]. Studies of the mutational landscape of PBL showed *MYC* translocation in about 50% of cases, as well as recurrent mutations in the RAS/RAF and JAK/STAT signaling pathways [14, 15]. Our patient’s transformed PBL showed *IGH*::*MYC* fusion and *BRAF* V600E mutation, both well-known in PBL. *IRF4* rearrangement is not typically detected in PBLs, and there has been only one report of PBL with *IRF4* rearrangement to date. The patient was 14-year-old girl who showed coexisting *IRF4* and *MYC* rearrangements as in our case, but the fusion partner of *IRF4* was *IGK* [16]. Although *IRF4* rearrangement is rare in PBLs, amplification of *IRF4* was detected in up to 29% of cases and was functionally significant according to a study by Frontzek et al. [14]. Our case is unique in that it demonstrated coexisting *IRF4* and *MYC* rearrangements; this is the second such report among published cases of PBLs to date.

*MYC* translocation has also been reported in cases of transformed PBL from t(14;18)-positive FL [7, 9]. In a study by Ouansafi et al. [9], *BCL2* rearrangement was detected in both the initial FL and the transformed PBL, whereas *MYC* rearrangement was detected only in the transformed PBL. In our case, *MYC* rearrangement was detected by both NGS and FISH in the PBL. However, *MYC* rearrangement was detected only by FISH in some FL cells, suggesting that the FL had a subclonal *MYC* rearrangement. *MYC* rearrangement is uncommon in FL; in one study, such rearrangement was reported in up to 1.9% of FLs, including grade 1–2 and grade 3A FL, with or without *BCL2* rearrangement [17]. However, the prognostic significance of *MYC* rearrangement in FL remains unclear [17].

In our case, a t(14;18)-negative FL clone with *CREBBP*, *STAT6*, *BCL6*, and *CD79B* mutations (common progenitor in Fig. 2B), divergently evolved into FL with additional *TNFRSF14*, *KLHL6*, *CD79A*, and *EZH2* mutations and PBL (Fig. 2B). The transformation to PBL occurred after gaining the *BRAF* V600E mutation and *IGH*::*IRF4* fusion, in addition to clonal expansion of *MYC*-rearranged cells. Considering the short interval until the PBL developed, the transformation might have occurred early in the disease progress. The transformed PBL subpopulation may have rapidly expanded after rituximab administration. Furthermore, the initial t(14;18)-negative FL in our case was similar to dFL or *BCL2-R*−negative CD23^+^ FCL in that it showed CD23 expression with *STAT6* and *CREBBP* co-mutation. However, aggressive behavior is unusual for dFL or *BCL2-R*−negative CD23^+^ FCL; in this case, it may be partly explained by *MYC*-rearranged subclones and *CDKN2A* copy number loss at initial presentation.

Here, we present the first case of transformation of t(14;18)-negative FL into PBL, with supporting genetic evidence. This study broadens the spectrum of histological transformation of t(14;18)-negative FL and emphasizes its biological and clinical heterogeneity. Our case also demonstrated divergent clonal evolution of a common progenitor t(14;18)-negative FL into FL and an aggressive lymphoma. Such clonal evolution is a well-known phenomenon observed in classic FLs when relevant genetic mutations are present, but little is known regarding its occurrence in t(14;18)-negative FL.

## Data Availability

The datasets used and/or analyzed during the current study are available from the corresponding author on reasonable request.

## Abbreviations

*BCL2*-R: *BCL2* rearrangement
CT: Computed tomography
DLBCL: Diffuse large B-cell lymphoma
EBV: Epstein-Barr virus
FDG: Fluorine-18 labeled fluorodeoxyglucose
FISH: Fluorescence in situ hybridization
FL: Follicular lymphoma
HIV: Human immunodeficiency virus
ITAM: Immunoreceptor tyrosine-based activation motif
LDH: Lactate dehydrogenase
LN: Lymph node
NGS: Next-generation sequencing
PBL: Plasmablastic lymphoma
PET: Positron emission tomography
